# Brain Metastases from Lung Cancer Show Increased Expression of DVL1, DVL3 and Beta-Catenin and Down-Regulation of E-Cadherin

**DOI:** 10.3390/ijms150610635

**Published:** 2014-06-13

**Authors:** Anja Kafka, Davor Tomas, Vili Beroš, Hrvoje Ivan Pećina, Martina Zeljko, Nives Pećina-Šlaus

**Affiliations:** 1 Laboratory of Neuro-Oncology, Croatian Institute for Brain Research, School of Medicine, University of Zagreb, Salata 12, 10000 Zagreb, Croatia; E-Mails: anja.kafka@mef.hr (A.K.); martina.zeljko2@gmail.com (M.Z.); 2 Department of Biology, School of Medicine, University of Zagreb, Salata 3, 10000 Zagreb, Croatia; 3 Ljudevit Jurak Department of Pathology, University Hospital “Sisters of Charity”, 10000 Zagreb, Croatia; E-Mail: davor.tomas@kbcsm.hr; 4 Department of Pathology, School of Medicine, University of Zagreb, Salata 10, 10000 Zagreb, Croatia; 5 Department of Neurosurgery, University Hospital “Sisters of Charity”, 10000 Zagreb, Croatia; E-Mail: viliberos@gmail.com; 6 Department of Radiology, University Hospital “Sisters of Charity”, 10000 Zagreb, Croatia; E-Mail: hipecina@gmail.com; 7 Department of Internal Medicine, “Merkur” University Hospital, 10000 Zagreb, Croatia

**Keywords:** Dishevelled-1 (DVL1), Dishevelled-3 (DVL3), E-cadherin (CDH1), beta-catenin (CTNNB1), brain metastases, lung cancer, immunostaining, loss of heterozygosity

## Abstract

The susceptibility of brain to secondary formation from lung cancer primaries is a well-known phenomenon. In contrast, the molecular basis for invasion and metastasis to the brain is largely unknown. In the present study, 31 brain metastases that originated from primary lung carcinomas were analyzed regarding over expression of Dishevelled-1 (DVL1), Dishevelled-3 (DVL3), E-cadherin (CDH1) and beta-catenin (CTNNB1). Protein expressions and localizations were analyzed by immunohistochemistry. Genetic alterations of E-cadherin were tested by polymerase chain reaction (PCR)/loss of heterozygosity (LOH). Heteroduplex was used to investigate mutations in beta-catenin. DVL1 and DVL3 showed over expression in brain metastasis in 87.1% and 90.3% of samples respectively. Nuclear staining was observed in 54.8% of cases for DVL1 and 53.3% for DVL3. The main effector of the Wnt signaling, beta-catenin, was up-regulated in 56%, and transferred to the nucleus in 36% of metastases. When DVL1 and DVL3 were up-regulated the number of cases with nuclear beta-catenin significantly increased (*p* = 0.0001). Down-regulation of E-cadherin was observed in 80% of samples. Genetic analysis showed 36% of samples with LOH of the *CDH1*. In comparison to other lung cancer pathologies, the diagnoses adenocarcinoma and small cell lung cancer (SCLC) were significantly associated to *CDH1* LOH (*p* = 0.001). Microsatellite instability was detected in one metastasis from adenocarcinoma. Exon 3 of beta-catenin was not targeted. Altered expression of Dishevelled-1, Dishevelled-3, E-cadherin and beta-catenin were present in brain metastases which indicates that Wnt signaling is important and may contribute to better understanding of genetic profile conditioning lung cancer metastasis to the brain.

## 1. Introduction

Metastasis is a highly selective process conditioned by the genetic profile of the original tumor as well as by organ environment. The susceptibility of the brain to secondary formation from lung cancer primaries is a well-known phenomenon. In contrast, the molecular basis for invasion and metastasis to the brain is largely unknown. Reports indicate that specific set of genes mediate metastasis to the brain according to discrete changes [1,2].

The work presented in this paper focused on the analysis of different expression levels of Dishevelled-1 (DVL1), Dishevelled-3 (DVL3), beta-catenin (CTNNB1) and E-cadherin (CDH1) in brain metastases that originated from primary lung carcinomas. All the molecules studied are key players in the classical Wnt signaling pathway which has today been established as one of the basic cellular pathways and whose misregulation plays an important role in tumorigenesis. Initiation of the Wnt signaling cascade depends on the presence of Wnts, glycoproteins that bind to receptors called frizzled. Thus, activated, the receptors recruit Dishevelleds at cytoplasmic membrane and this interaction also pulls axin from the beta-catenin destruction complex to the plasma membrane. In this fashion the complex is inactivated [3] leading to the stabilization of cytosolic beta-catenin. Stabilized beta-catenin enters the cell nucleus and associates with T-cell factor (TCF)/lymphoid enhancer factor (LEF) transcription factors, which leads to the transcription of Wnt-target genes such as c-myc, matrix metalloproteinase (MMP)7, cyclin D1, *etc.* [4,5,6,7,8,9,10]. In the absence of factors that activate Wnt signaling, the beta-catenin destruction complex that consists of adenomatous polyposis coli (APC)-Axin and glycogen synthase kinase 3 (GSK3) binds to beta-catenin with subsequent beta-catenin phosphorylation, ubiquitination, and degradation by proteasomes. In this scenario beta-catenin/TCF/LEF target genes are repressed [4,5,11]. However, mutations of the complex APC-Axin and GSK3 can also results in the translocation of beta-catenin to the nucleus ultimately leading to oncogenic transformation and progression.

The cellular function of human Dishevelled genes has not been completely elucidated yet. Nevertheless, Dishevelleds (DSH or DVL) are considered to be the central hub of Wnt signaling and are all multifunctional phosphoproteins that have been shown to shuttle between the cytoplasm and the nucleus [12,13,14]. The reported data on Dishevelleds’ role in tumorigenesis is controversial, although it has been demonstrated that they are over expressed in several types of cancers including lung cancer [15,16,17,18,19].

Of particular interest is a process very much involved in invasion and metastasis of tumors—the so called Epithelial-to-Mesenchymal Transition (EMT), where noninvasive tumor cells acquire motility and ultimately disseminate to places distant from the primary site. It has been shown that the Wnt signaling has a particularly tight link with EMT. For example, the nuclear translocation of beta-catenin can induce EMT [6,20,21] by activating the transcriptional repressors Snail and Slug that suppress E-cadherin expression [6,22,23,24]. Another important finding is that lymphoid enhancer factor 1 (LEF1) when over expressed leads to enhanced tumor invasiveness and induces EMT [2,20,25,26].

Besides being the main signaling effector molecule of the pathway, beta-catenin is also involved in the cellular architecture. It is bound to E-cadherin and is an essential component of adherens junctions. In this respect, it is important to remember that the most prominent feature of EMT is the destruction of adherens junctions and the loss of expression of the cell-cell adhesion molecule E-cadherin.

Our hypothesis is that molecular components of the Wnt pathway, Dishevelleds, E-cadherin and beta-catenin, play important roles in metastasis of lung cancer to the brain. We believe that their altered levels of expression contribute to the outline of molecular profile of distant brain metastases.

## 2. Results

DVL1 and DVL3 showed over expression in brain metastasis tissues in 87.1% and 90.3% of samples respectively. The staining was observed mainly in the cytoplasm and was diffusely or granularly distributed. Nevertheless, we also observed nuclear staining of DVL1 in 54.8% of cases and DVL3 in 53.3% of cases.

**Figure 1:**
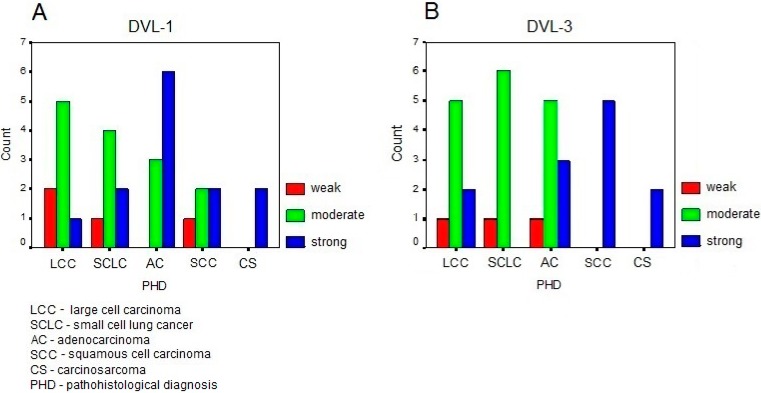
The distribution of the expression levels of the two proteins in metastases according to the starting point of specific type of the primary lung cancer. (**A**) Dishevelled-1 (DVL1); and (**B**) Dishevelled-3 (DVL3).

At the level of the complete brain metastasis sample, our analyses showed that there were 12.9% of samples demonstrating weak expression of DVL1; 45.2% moderate expression and 41.9% strong expression. Considering the expression levels of DVL3 in our total sample we observed 9.7% of samples with weak expression; 51.6% with moderate expression and 38.7% with strong expression. The normal levels of expression in healthy tissues were very weak. The expression levels of the two proteins in metastases, according to the starting points of the specific types of primary lung cancers are shown in Figure 1. For DVL1 the expression levels were not statistically different between the various pathological diagnoses (χ^2^ = 9.375; *df* = 8; *p* = 0.312). In contrast to this, the expression levels of DVL3 were statistically significantly different between the same pathological diagnoses (χ^2^ = 16.275; *df* = 8; *p* = 0.039).

Normal levels of E-cadherin staining were scored as +++, and the protein was located along the cell membrane or inside the cytoplasm. The investigation on E-cadherin showed that overall 80% of metastases had down-regulation of E-cadherin. Intense down-regulation was noticed in 52% of cases. The majority of samples (88.9%) with E-cadherin’s gross deletions (LOH) were accompanied with down-regulation of E-cadherin protein. All metastases originated from small cell lung cancer (SCLC), adenocarcinoma and carcinosarcoma showed E-cadherin down-regulation, while in large cell carcinoma and squamous cell carcinomas E-cadherin was down-regulated in 75% and 60% cases respectively. The expression levels of Dishevelleds were not associated to E-cadherin down-regulation (*p* = 0.856 for DVL1; *p* = 0.310 for DVL3).

Our next step was to detect whether the expression and cellular localization of beta-catenin, correlated with the expression levels of Dishevelleds and E-cadherin. We noticed that beta-catenin was up-regulated in 56%, and transferred to the nucleus in 36% of metastases. It is interesting to note that 40.9% of cases with up-regulated DVL1 expression had beta-catenin transferred to the nucleus. The percentage of metastases with up-regulated DVL3 that also exhibited nuclear staining of beta-catenin was 32%. When DVL1 was up-regulated the number of cases with nuclear beta-catenin significantly increased (McNemar test, *p* = 0.0001). The same trend was noticed when DVL3 was up-regulated (McNamar, test *p* = 0.0001).

Approximately 25% of metastases showed simultaneous nuclear localization of Dishevelleds and beta-catenin. Statistical analysis showed a significant correlation between the nuclear localization of beta-catenin and DVL1 (*p* = 0.05), but no correlation between nuclear DVL3 and beta-catenin (*p* = 0.261).

Only 4 primary lung cancers autologous to the brain metastases were available for the analysis. Three out of 4 primary lung cancers showed higher E-cadherin’s and lower beta-catenin’s expression levels than in the autologous brain metastases. All four primaries showed lower DVL1 expression levels when compared to the corresponding metastases, and 2 out of 4 primaries showed lower DVL3 levels.

**Figure 2:**
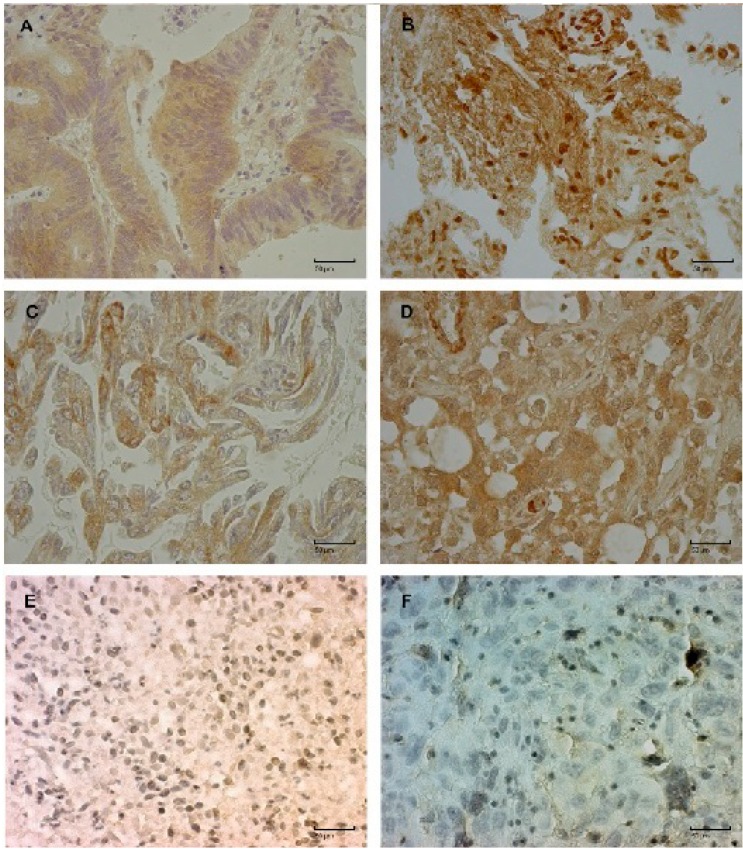
Brain metastasis samples immunohistochemically stained for the expression of DVL1, DVL3, E-cadherin and beta-catenin proteins. Strong expression of DVL1, (**A**) in cytoplasm; (**B**) in cytoplasm and nucleus; Strong expression of DVL3, (**C**) in cytoplasm; (**D**) in cytoplasm and nucleus; Nuclear expression of beta-catenin (**E**); and Loss of expression of E-cadherin protein (**F**). Original magnification, 400×; scale bar, 50 μm.

Detailed histopathological diagnosis, the origin of the lesions of metastasis sample and findings on the protein levels are presented in Table 1. Immunostaining of Dishevelleds 1 and 3 is demonstrated in Figure 2A–D and of beta-catenin protein in Figure 2E. Loss of E-cadherin’s expression is demonstrated in Figure 2F.

**Table 1:** The localization of the metastases, the polymorphic status for microsatellite markers used and genetic changes of the *CDH1* gene, expression levels of E-cadherin, beta-catenin, DVL1 and DVL3 proteins, and pathohistological diagnosis of the primary site.

Patient No.	Location	*CDH1* D16S752&D16S265&D16S398	E-Cadherin	Beta-Catenin	Dishevelled-1	Dishevelled-3	Primary Tumor
1	Cerebellum	LOH D16S265	0	C++ N+	++	+++	Large cell carcinoma
2	Cerebellum	HETERO	+++	C++	++	++	Large cell carcinoma
3	Frontal region	HETERO	++	0	+	++	Large cell carcinoma
4	Parietal region	HETERO	+++	C+	+++	++	Large cell carcinoma
5	Occipital region	HETERO	+	C++	++	+++	Large cell carcinoma
6	Frontal region	HETERO	+	N++	++	+	Large cell carcinoma
7	Occipital region	HETERO	++	C+N+++	++	++	Large cell carcinoma
8	Frontal region	HETERO	+	C++	+	++	Large cell carcinoma
9	Parietal region	HETERO	+	C+	++	++	SCLC
10	Frontal region	LOH D16S398	0	C+	++	++	SCLC
11	Parietal region	LOH all	0	C++N+++	++	++	SCLC
12	Parietooccipital region	LOH D16S265	++	C+	+++	++	SCLC
13	Parietal	ND	ND	ND	++	++	SCLC
14	Parietal	ND	ND	ND	+	+	SCLC
15	Cerebellum	ND	ND	ND	+++	++	SCLC
16	Cerebellum	MSI D16S265	0	C+	+++	+++	Adenocarcinoma
17	Temporal region	HETERO	+	C+	++	++	Adenocarcinoma
18	Parietal region	LOH D16S752	++	C+	+++	+++	Adenocarcinoma
19	PRFrontal region	NDLOH all	++++++	C+++C++N+	++++	++++	Adenocarcinoma
20	Temporal region	LOH D16S398	++	C+	++	++	Adenocarcinoma
21	PRCerebellum	NDLOH D16S752	++++	0C+	+++++	++++	Adenocarcinoma
22	Temporal	ND	ND	ND	+++	+	Adenocarcinoma
23	Frontoparietal	ND	ND	ND	++	++	Adenocarcinoma
24	Frontoparietal	ND	ND	ND	+++	++	Adenocarcinoma
25	Parietal region	LOH D16S398	0	C++	+	+++	Squamous cell carcinoma
26	Parietal region	HETERO	+++	C+N++	+++	+++	Squamous cell carcinoma
27	Parietal region	HETERO	+++	C+++	++	+++	Squamous cell carcinoma
28	Multiple metastases	HETERO	++	C++N+	++	+++	Squamous cell carcinoma
29	Cerebellum	HETERO	+	C+N++	+++	+++	Squamous cell carcinoma
30	PRTemporal region	NDHETERO	++++	C++C+++N+	+++++	++++++	Carcinosarcoma
31	PRParietal region	NDHETERO	++++	0C+	++++	+++++	Carcinosarcoma

LOH = loss of heterozygosity; MSI = microsatellite instability; HETERO = heterozygous samples; HOMO = homozygous samples; ND = not determined; 0 = no expression; + = weak expression; ++ = moderate expression; +++ = strong expression; C = cytoplasmic; N = nuclear localization; All = all three MS markers D16S752&D16S265&D16S398 showing LOH; SCLC = small cell lung cancer; PR = denotes expression in the autologous primary lung cancer.

The results of our genetic analysis indicate a total of 36% of the sample with LOH of the *CDH1* (E-cadherin) gene when assessed with 3 markers. The highest frequency of genetic changes was observed in metastases originating from primary sites with the diagnosis of lung adenocarcinoma and SCLC with 83.3% and 75% of changes, respectively, while squamous cell carcinoma (SCC) exhibited 25%, and large cell carcinoma (LCC) 12.5% of LOHs. In comparison to other lung cancer pathologies, the diagnoses adenocarcinoma and SCLC were significantly associated to E-cadherin genetic changes with χ^2^ = 10.364; *df* = 1; *p* = 0.001. A type of genomic instability—microsatellite instability (MSI) was detected in one metastasis originated from adenocarcinoma. The results of heteroduplex analysis of exon 3 did not show samples with additional bands which indicated that mutational hot-spot of beta-catenin, was not targeted in our metastasis sample.

## 3. Discussion

Our results on DVL1, DVL3, beta-catenin and E-cadherin bring novel insights on the Wnt signaling role in brain metastases that originated from primary lung carcinomas. We investigated the combination of Wnt key molecules in our collected tumor sample because Wnt signaling is involved in EMT and metastasis formation [27]. The results of investigations of DVL1 and DVL3 protein levels showed over expression in brain metastasis tissues in 87.1% and 90.3% of samples respectively. The involvement of DVL members in lung cancer is still very much unknown, particularly the association between changed expression and the pathohistological type of lung cancer from which the metastasis originated. Moreover, the correlation with tumor prognosis is still not established. Only several papers report the over expression of Dishevelleds in primary lung cancers. Uematsu *et al.* [18] report on DVL3 over expression in non-small cell lung cancer. They showed that 75% NSCLC tumors (¾ squamous and ¾ adeno) had DVL3 overexpression. They imply that these events, upstream of beta-catenin, are critical for activation of Wnt signaling. Surprisingly these authors did not detect expression of DVL1 or DVL2 which is different from our findings on the presence of DVL1 over expression.

Li *et al.* [28] discovered that DVL3 mRNA levels are significantly higher in pleural effusions from patients with adenocarcinoma and offered this finding as a marker for pleural micrometastasis.

The findings that our investigation builds on are brought by Zhao *et al.* [16] and Wei *et al.* [29] who found that Dishevelled family proteins are over expressed in primary lung cancers. Zhao *et al.* [16] found positive expression of DVL1 and DVL3 in 45.1% and 48% of non small cell lung cancer (NSCLC) (squamous cell carcinoma (SCC) and adenocarcinoma). The expression levels of Dishevelleds were significantly higher in adenocarcinomas than squamous cell carcinomas.

Wei *et al.* [29] showed that the positive expression rate in primary NSCLC tumors was 53.1% for total DVL, 36.3% for DVL1, 36.3% for DVL2 and 41.6% for DVL3. They also demonstrated that the positive expression rates of Dishevelleds were higher in tumor stages III and IV. Nodal metastases included in their study also showed higher expression levels of DVL1 and DVL3 than primary growths. Our findings on very elevated levels of Dishevelleds in brain metastases represent a logical continuation of the findings reported by these authors in primary lung carcinomas. Moreover, Wei *et al.*’s [29] data on the rise of Dishevelleds’ expression from primary (53.1%) to nodal metastases (87.2%) is additionally confirmed by our analysis that shows that 85.2% of Dishevelleds’ over expression is present when brain metastases are the final destination.

So far it has been reported that DVLs are over expressed in various tumors types. The results from Nagahata *et al.* [15] suggest that amplification and up-regulation of the *DVL1* gene are involved in breast carcinogenesis, especially in the acceleration of tumor growth. The involvement of DVLs in invasive ductal carcinoma of the breast was also reported by Prasad *et al.* [30]. Mizutani *et al.* [31] got similar results in prostate cancer. Their sample of 20 primary prostate cancer showed significant over expression of DVL1 (65%) and correlation to beta-catenin’s expression. Pulvirenti *et al.* [14] showed that DVL2 is over expressed in human high-grade gliomas. Results from Okino *et al.* [32] demonstrate increased expression of DVL1 in over two thirds of primary cervical squamous cell cancers when compared to corresponding non-cancerous uterine squamous cell tissues. All these data collectively indicate that amplification and increased expression of the *DVL* genes may play important role in the development of a portion of human cancers through derangement of the Wnt signaling pathway.

Although reports indicate DVLs location in the nucleus is only sporadic [29], our study on brain metastases did not show this feature. We found nuclear staining of DVL1 proteins in 54.8% of cases and of DVL3 in 53.3%. It is known that DVLs interact with the wide range of protein partners in both the cytoplasm and the nucleus [13]. Recently collected data [13,33,34,35] suggest that there are two cellular pools of DVLs: one that translocates to the nucleus, while the other remains in the cytoplasm. While it is not clear how it is regulated, when DVL is located in the nucleus it is known to interact with beta-catenin [13,36,37,38]. Our data support these findings by demonstrating that 25% of metastases simultaneously exhibit nuclear localization of Dishevelleds and beta-catenin.

The majority of metastases had down-regulation of E-cadherin expression with intense down-regulation in 52% of cases. Similar results were reported by McDonald *et al.* [39] who also detected lower E-cadherin expression in metastatic lesions. Additionally, Saad *et al.* [40] demonstrated that loss of E-cadherin in patients with adenocarcinoma and squamous cell carcinoma (SCC) of the lung is significantly associated with increased risk of developing brain metastases. The results of other authors investigating E-cadherin in brain metastasis [41,42,43] collectively demonstrate that E-cadherin is constantly expressed in metastatic deposits. E-cadherin was expressed in the majority of our sample too, but contrary to the above studies, we observed different levels of its expression. E-cadherin’s expression in primary lung cancers is down-regulated in invasive component of 62% adenocarcinoma [44], and in another study in 72% of SCC and adenocarcinoma [45]. In 2 investigated cases of carcinosarcoma 70% of cells showed E-cadherin’s down-regulation [46].

Recent studies [10] also show that DVLs are involved in tumor metastasis. Results from Shi *et al.* [47] indicate that specific protein domains shared by Dishevelleds and axin 1 (PDZ-RGS3 domains) enhance signals generated by Wnt canonical pathway which plays important role in EMT and tumor migration.

Nuclear location of beta-catenin is an indicator of its acquisition of oncogenic activity. In our study, beta-catenin was up-regulated in 56%, and transferred to the nucleus in 36% of metastases. It is interesting to note that when DVL1 was up-regulated the number of cases with nuclear beta-catenin significantly increased (*p* = 0.0001). Additionally, when DVL3 was up-regulated, there was a significant number of cases with beta-catenin nuclear staining (*p* = 0.0001). A study by Guldur *et al.* [48] showed 66.7% of LCC samples, 53.4% of SCC and 42% of adenocarcinoma with positive beta-catenin staining, while all of their SCLC samples showed reduced beta-catenin staining. A study by Rodriguez-Salas *et al.* [49] showed cytoplasmic hyperexpression in 28% of SCLC and no nuclear beta-catenin staining. Only two samples were reported for carcinosarcoma staining of beta-catenin [46] and 70% of cells expressed beta-catenin with the strong intensity.

In heteroduplex analysis, the conformational properties of the double stranded molecules are used to distinguish different base pairing (*i.e.*, mutations). Annealing of mutant DNA to wild type probe gives duplexes with one or more mismatched bases (heteroduplexes). Mismatching causes the double helix to take on a conformation which retards its mobility during electrophoresis [50]. The results of our heteroduplex analysis did not demonstrate mutations of beta-catenin suggesting that mutations in exon 3 of the *CTNNB1* gene are not associated to brain metastasis process. This finding is not unusual since many investigations [51,52,53] collectively demonstrated that mutation of *APC*, *AXIN2* or beta-catenin are rarely seen in lung cancer [53,54] or are mutually exclusive, which is supported by our results. Uematsu *et al.* [19] also failed to detect mutations in exon 3 of beta-catenin in mesothelioma. Therefore nuclear beta-catenin accumulation may be a result of mechanisms other than mutation.

Since there are no known mutations of beta-catenin in lung cancer it seems that alterations of the upstream effectors of beta-catenin’s degradation are responsible for the Wnt activation. The over expression of Dishevelleds act upstream of the destruction complex stealing axin from it and thus disrupting it or preventing its proper formation. This event enables cytoplasmic accumulation of beta-catenin and its translocation to the nucleus which is also observed in our study. The underlying molecular mechanisms suggested by our study are that Dishevelleds over expression enhance transcriptional activity of beta-catenin.

We are aware that our study was carried out on a relatively small number of brain metastases and, therefore, all conclusions made on this data set need to be tempered with caution. It is, however, important to note that despite the small number of cases analyzed, we recorded significant associations between DVL3 expression levels between the metastases originating from various primary tumors, upregulation of DVL1 and DVL3 and nuclear beta-catenin staining and also association of *CDH1* genetic changes and diagnoses adenocarcinoma and SCLC. What needs to be taken into account is the fact that the overall number of investigated cases reported in medical literature so far still remains shockingly small—after a comprehensive search of the available literature we found just 270 cases of lung cancer investigated for DVL1 and DVL3, and of these there was no data on potential brain metastasis.

Although we are aware that it would be very interesting to draw comparisons between the primary lung cancer and the metastatic deposits, unfortunately it was not possible to attain the samples from the primary lung cancer except in 4 cases. Nevertheless, many authors performed analyses on primary lung cancer and report on the expression levels. Obviously, what needs to be done in the future is to analyze primary tumors and metastases in the same patient.

Our previous investigations on brain metastases showed the involvement of yet another important component of Wnt signaling, the *APC* gene [55]. We found that 55% of metastases from lung carcinoma had LOH of this tumor suppressor gene. Three adenocarcinomas, 1 large cell carcinoma, 1 small cell carcinoma, and 1 squamous cell carcinoma harbored gross deletions of *APC* gene. Although LOH of the *APC* gene is a common finding in lung cancer, point mutations of this tumor suppressor have not been frequent [54]. It is therefore interesting to report our finding of somatic mutation in exon 15 of the *APC* gene detected in one case of brain metastase derived from SCLC by direct DNA sequencing of the metastasis and autologous lymphocyte samples. The substitution was at position 5883 G–A in the metastasis tissue [56].

The biologic spectrum of metastases is unfortunately wide, heterogenic and difficult to predict, resulting in poor prognosis. Their proliferative activity and invasion are important characteristics that should be considered in diagnostics and prognosis. Why is the metastasis process from the lung so fast, particularly in comparison with the breast and the other primary locations? The velocity of metastasis establishment differs depending on the primary site and may therefore differ according to the genes involved. However, relevant information on the genes that are crucial in the metastatic process has not yet been utilized in diagnostics, prognostics, or therapeutic purposes. Novel findings point out the importance of chemokines and their receptors in brain metastasis formation [57]. Lung cancer is one of the most common malignancies and is the leading cause of cancer death in industrialized countries. Novel therapies aimed at inhibition of metastasis process based on better understanding of the biology of brain metastases will offer improvement in diagnosis and treatment of this disease.

## 4. Experimental Section

### 4.1. Tumor Specimen

Samples of 31 brain metastases from lung cancer, together with autologous blood samples were collected from the Department of Pathology and Department of Neurosurgery University Hospital “Sisters of Mercy”, Zagreb, Croatia. Using the magnetic resonance imaging (MRI) metastasis lesions were found in different cerebral regions, most frequently in parietal and frontal regions and cerebellum, with the surrounding zone of perifocal oedema. During the operative procedure the metastasis was maximally reduced using a microneurosurgical technique. The patients had no family history of brain tumors and did not receive radio- or chemotherapy before metastasis excision. All metastases were studied by pathologists and classified according to the WHO criteria [58]. There were 8 large cell carcinoma (LCC), 7 small cell lung cancer (SCLC), 9 adenocarcinoma (AC), 5 squamous cell carcinoma (SCC) and 2 carcinosarcoma (CS) cases. The metastasis tissues for DNA extraction were frozen in liquid nitrogen and transported to the laboratory, where they were immediately transferred at −75 °C. The peripheral blood samples were collected in EDTA and processed immediately.

Twenty-two patients were male, and 9 were female. The age of patients varied from 46 to 81 (mean age 60.48, median 60 years). The mean age at diagnosis for males was 59.95, and for females 61.4 years.

The local Ethical Committee approved our study, grant 1.2.1.19., and patients gave their informed consent.

### 4.2. Immunohistochemistry

Immunohistochemistry was performed in order to establish the levels of expression and cellular localization of Dishevelled-1, Dishevelled-3, beta-catenin and E-cadherin proteins. The samples were fixed in formalin, embedded in paraffin, sliced into 4-μm thick sections, and then fixed onto capillary gap microscope slides (DakoCytomation, Glostrup, Denmark). Sections were immunostained using streptavidin horseradish peroxidase/DAB (3,3'-diaminobenzidine) (Dako REAL™, EnVision™, Glostrup, Denmark). Briefly, sections were deparaffinized and rehydrated and then microwaved twice for 3 min at 700 W in citrate buffer and once for 4 min at 350 W to unmask epitopes. To block endogenous peroxidase activity, cells were fixed in methanol with 3% H_2_O_2_. Non-specific binding was blocked by incubating samples with goat serum for 30 min at 4 °C. Next, the primary antibodies were applied for 30 min at room temperature. The antibodies used for Dishevelled protein detection were: polyclonal rabbit anti-human DVL1 (diluted 1:50), Abcam, Cambridge, UK and monoclonal mouse anti-human DVL3 (diluted 1:50), Santa Cruz Biotehnology, Dallas, TX, USA. For E-cadherin protein detection monoclonal mouse anti-human E-cadherin NCH-38 (diluted 1:100), and for beta-catenin monoclonal mouse anti-human antibody (diluted 1:200), both Dako Corporation, Carpinteria, CA, USA, were used.

Slides were then washed three times in phosphate-buffered saline (PBS)/goat serum, and secondary LINK antibody was applied for 1 h at 4 °C. Slides were again washed three times in PBS/goat serum and were incubated with substrate chromogen solution (EnVision™, Dako REAL™) for 30 s.

Negative controls underwent the same staining procedure but without incubating samples with the primary antibodies. The frontal cortex of a normal brain, normal human placenta and normal bronchial epithelia served as positive controls. Antibody labeling was analyzed by three independent and blinded observers using an Olympus BH-2 microscope. No expression or very weak expression was labeled as 0/+, moderate expression as ++, and strong expression as +++. Two hundred cells of each sample were analyzed.

### 4.3. Genetic Analyses of E-Cadherin and Beta-Catenin

For the genomic DNA extraction approximately 0.5 g of metastasis tissue was homogenized with 1 mL extraction buffer (10 mM Tris–HCl, pH 8.0; 0.1 M EDTA, pH 8.0; 0.5% sodium dodecyl sulfate) and incubated with proteinase K (100 µg/mL; Sigma-Aldrich, St. Louis, MO, USA; overnight at 37 °C). Phenol chloroform extraction and ethanol precipitation followed. Blood was used to extract leukocyte DNA. Five milliliter of blood was lysed with 7 mL distilled water and centrifuged (15 min/5000× *g*). The pellet was then processed as for DNA extraction from the tissue samples.

#### 4.3.1. Polymerase Chain Reaction (PCR)

PCR amplification of microsatellite markers for the *CDH1* (E-cadherin) gene was used to test loss of heterozygosity (LOH) in brain metastases. Allelic loss at the *CDH1* locus was assessed using 3 highly polymorphic microsatellite markers—D16S265, D16S398 and D16S752, which map to 16q21–22.1 [59]. D16S265 and D16S398 are CA dinucleotide repeat polymorphisms and the D16S752 polymorphic region is a tetranucleotide GATA polymorphism (GATA51G03). All markers were amplified in a total volume of 25 µL, each primer 5 pmol, 200 ng DNA, 2.5 µL 10× buffer II, 1.5 mM MgCl_2_, 2.5 mM of each dNTP, 0.5 U Taq (Promega, Fitchburg, WI, USA). PCR conditions for D16S265, D16S398: initial denaturation 1 min/95 °C; denaturation, 30 s/95 °C; annealing, 1 min/55 °C; extension, 30 s/72 °C; final extension, 72 °C/7 min; 30 cycles. PCR conditions for D16S752: initial denaturation 3 min/96 °C; denaturation, 30 s/96 °C; annealing, 35 s/55 °C; extension, 30 + 1 s/72 °C; final extension, 72 °C/10 min; 30 cycles.

Primers for D16S752: 5'-AATTGACGGTATATCTATCTGTCTG-3'; and 5'-GATTGGAGGAGGGTGATTCT-3'; for D16S265: 5'-CCAGACATGGCAGTCTCTA-3' and 5'-AGTCCTCTGTGCACTTTGT-3'; and for D16S398: 5'-CTTGCTCTTTCTAAACTCCA-3' and 5'-GAAACCAAGTGGGTTAGGGTC-3'.

PCR products were analyzed on 2% agarose gels, length of the D16S752 repeat was 102–126 bp, length of the D16S265 repeat was 89–117 bp, and length of the D16S398 repeat was 180–196 bp.

#### 4.3.2. Loss of Heterozygosity, Microsatellite Instability (MSI)

Absence or significant decrease in the intensity of one polymorphic allele in metastasis compared to the heterozygous autologous blood sample was considered as LOH of *CDH1* gene. The samples were electrophoresed on Spreadex gels EL 300 in the SEA 2000 submarine electrophoresis apparatus (Elchrom Scientific, Cham, Switzerland) at 120 V. Temperature of the running buffer was kept constant at 55 °C. The samples were stained with SyberGold (Molecular Probes, Leiden, The Netherlands). All the PCR experiments were repeated twice and the LOHs were confirmed. The samples with LOHs were additionally electrophoresed on 15% polyacrylamide gels stained with silver.

MSI is a type of genomic instability indicating impaired cellular mismatch repair. Samples demonstrating MSI have bands on different positions in comparison to bands of autologous blood tissue due to a defect in the replication/repair machinery in tumor cells.

#### 4.3.3. Heteroduplex Analysis

Exon 3 of the *CTNNB1* (beta-catenin) gene was screened for mutations. Heteroduplexes were formed by heating 3 µL of PCR products (tumor mixed with normal DNA) at 95 °C for 3 min, followed by incubation on ice for 20 min. About 3 µL of each sample was mixed with 7 µL of mixture of formamide and 10 mM NaOH (1:100) prior to loading to a gel. The electrophoresis was performed on the GMA (Gene Mutation Analysis) gels in the SEA 2000 submarine electrophoresis apparatus (Elchrom scientific, Cham, Switzerland). The temperature of the running buffer was kept constant at 9 °C.

### 4.4. Statistical Analysis

All individuals were analyzed for the following features: pathohistological diagnosis (PHD) status, sex, age, *CDH1* genetic changes, E-cadherin, Dishevelled 1, 3 and beta-catenin protein expression levels and localizations. Differences in the frequencies of the analyzed features were tested with the Pearson χ^2^ test employing Yates correction when appropriate and McNamar’s test. All statistical evaluations were performed using the SPSS statistical package, version 14.0 (SPSS Inc., Chicago, IL, USA).

## 5. Conclusions

Our findings suggest that altered expressions of Dishevelled-1, Dishevelled-3, E-cadherin and beta-catenin were present in brain metastases from lung cancer which indicates that Wnt signaling may be essential for the progression of lung cancer.
